# Renal-function change after transjugular intra-hepatic portosystemic shunt placement and its relationship with survival: a single-center experience

**DOI:** 10.1093/gastro/goaa081

**Published:** 2020-12-03

**Authors:** Min Lang, Angela L. Lang, Brian Q. Tsui, Weiping Wang, Brian K. Erly, Bo Shen, Baljendra Kapoor

**Affiliations:** 1 Department of Radiology, Massachusetts General Hospital, Harvard Medical School, Boston, MA, USA; 2 Department of Anesthesia, Critical Care, and Pain Management, Massachusetts General Hospital, Harvard Medical School, Boston, MA, USA; 3 Department of Radiology, UCLA Medical Center, Los Angeles, CA, USA; 4 Department of Radiology, Mayo Clinic, Jacksonville, FL, USA; 5 Colorado School of Public Health, Aurora, Colorado, USA; 6 The Inflammatory Bowel Disease Center at Columbia, Columbia University Irving Medical Center, New York, NY, USA; 7 Division of Vascular and Interventional Radiology, Imaging Institute, Cleveland Clinic, Cleveland, OH, USA

**Keywords:** TIPS, transjugular intra-hepatic portosystemic shunt, portal hypertension, renal function, renal failure, mortality

## Abstract

**Background:**

The effect of transjugular intra-hepatic portosystemic shunt (TIPS) placement on renal function and the correlation of post-TIPS Cr with mortality remain unclear. This study aimed to assess the effect of TIPS placement on renal function and to examine the relationship between post-TIPS Cr and mortality risk.

**Methods:**

A total of 593 patients who underwent *de novo* TIPS placement between 2004 and 2017 at a single institution were included in the study. The pre-TIPS Cr level (T0; within 7 days before TIPS placement) and post-TIPS Cr levels, at 1–2 days (T1), 5–12 days (T2), and 15–40 days (T3), were collected. Predictors of Cr change after TIPS placement and the 1-year mortality rate were analysed using multivariable linear-regression and Cox proportional-hazards models, respectively.

**Results:**

Overall, 21.4% of patients (*n *=* *127) had elevated baseline Cr (≥1.5 mg/dL; mean, 2.51 ± 1.49 mg/dL) and 78.6% (*n *=* *466) had normal baseline Cr (<1.5 mg/dL; mean, 0.92 ± 0.26 mg/dL). Patients with elevated pre-TIPS Cr demonstrated a decrease in post-TIPS Cr (difference, −0.60 mg/dL), whereas patients with normal baseline Cr exhibited no change (difference, <0.01 mg/dL). The 30-day, 90-day, and 1-year mortality rates were 13%, 20%, and 32%, respectively. Variceal bleeding as a TIPS-placement indication (hazard ratio = 1.731; *P *=* *0.036), higher T0 Cr (hazard ratio = 1.834; *P *=* *0.012), and higher T3 Cr (hazard ratio = 3.524; *P *<* *0.001) were associated with higher 1-year mortality risk.

**Conclusion:**

TIPS placement improved renal function in patients with baseline renal dysfunction and the post-TIPS Cr level was a strong predictor of 1-year mortality risk.

## Introduction

Renal dysfunction is a common and serious problem in patients with advanced liver disease [1–4]. Patients with cirrhosis and portal hypertension often develop circulatory dysfunction characterized by disturbances in systemic and renal hemodynamics [5]. Approximately 20% of patients hospitalized for cirrhosis develop acute renal injury and the presence of renal impairment indicates a poor prognosis [6–8]. In fact, the presence of renal failure increases mortality risk from 32% to 65% in critically patients with cirrhosis [9].

Placement of a transjugular intra-hepatic portosystemic shunt (TIPS) is commonly performed for patients with various complications of portal hypertension [10–12]. Several studies have shown that TIPS placement not only decreases the risk of variceal bleeding and reduces ascites, but also improves renal function [11, 13–16]. Although the pre-procedural creatinine (Cr) level is a component of the MELD (Model for End-stage Liver Disease) score and is known to be a predictor of mortality [17], it is not clear how a change in Cr after TIPS placement is related to mortality and whether those with baseline renal dysfunction exhibit a greater degree of benefit. The potential effect of TIPS placement on renal function is clinically important and warrants further investigation [18–20]. Therefore, the aim of this study was to investigate the effect of TIPS placement on renal function and to examine the relationship between the post-TIPS Cr level and mortality in a large cohort of patients.

## Patients and methods

This retrospective study was approved by the Cleveland Clinic Review Board (protocol 14–793) with a waiver of informed consent and patient privacy was ensured in compliance with the Healthcare Information Portability and Accountability Act. All cases were identified through an ICD-9 code search of Cleveland Clinic’s electronic medical records and were verified for accuracy manually. All procedures and practices were in accordance with the Declaration of Helsinki.

### Patients

Inclusion criteria for the study included *de novo* TIPS placement with covered stent-graft (Viatorr; Gore & Associates; Flagstaff, AZ, USA) at the Cleveland Clinic between 2004 and 2017, as well as measures of pre-TIPS and post-TIPS Cr levels. Exclusion criteria included failure or reversal of the TIPS procedure, nonfunctioning TIPS at follow-up, and patients diagnosed with end-stage renal disease requiring dialysis. A total of 593 consecutive patients were included in this retrospective study.

Previous studies have demonstrated that a Cr level of 1.5 mg/dL is an important threshold value for managing acute kidney injury and predicting outcomes in hospitalized patients with cirrhosis and ascites [21, 22]. Therefore, the patients in our cohort were subdivided into those with normal renal function at baseline (Cr <1.5 mg/dL) and those with renal dysfunction at baseline (Cr ≥1.5 mg/dL) for sub-analysis.

### TIPS procedures

All TIPS procedures were performed using the previously described and standard technique [23]. In all patients, venograms were obtained using CO_2_ or contrast medium (Ultravist 300, Optiray 240, Isovue 300, Visipaque 320, Omnipaque 300, and CO_2_; Bayer HealthCare LLC, Whippany, NJ, USA). Portosystemic gradient (PSG) values were measured before and after the procedure. Variceal embolization was performed only in patients with persistent filling of the varices with antegrade flow after TIPS placement.

### Study variables

To determine the effect of TIPS placement on renal function, serum Cr levels were collected at four time points if available: (i) 0–7 days before the procedure (T0); (ii) 1–2 days after the procedure (T1); (iii) 5–12 days after the procedure (T2); and (iv) 15–40 days after the procedure (T3). These time periods were chosen to assess the temporal effect of TIPS placement on renal function, as it may take several days for the Cr level to stabilize after the procedure.

Demographic data were obtained from the electronic-medical-record system, including patient age at the time of TIPS placement, sex, race, history of encephalopathy, history of hypertension, etiology of liver disease, indication for TIPS creation, emergency TIPS creation, and date of death if applicable. The indication for TIPS placement was determined from pre-TIPS hepatology and interventional-radiology consultation reports, endoscopic reports, and imaging studies.

### Statistical analysis and outcome measures

Statistical analysis was performed using the computing environment R (Vienna, Austria) [24]. Paired *t*-test was used to compare the changes in Cr levels from T0 to T1, from T0 to T2, and from T0 to T3. *P*-values were adjusted for multiple comparisons based on Holm’s method.

Multivariable linear regression was used to assess the effects of age, sex, race, history of encephalopathy, etiology of liver disease, indication for TIPS placement, emergency TIPS placement, pre-TIPS (T0) Cr level, post-TIPS (T3) Cr level, and change in PSG on the changes in Cr levels from T0 to T3.

Cox proportional-hazards regression was used to assess the effects of age, sex, race, history of encephalopathy, etiology of liver disease, indication for TIPS placement, emergency TIPS placement, pre-TIPS (T0) Cr level, post-TIPS (T3) Cr level, and changes in PSG on the 1-year post-TIPS mortality rate. Because T3 and T0 were associated with each other, an interaction term between the two variables was included in the regression model.

Statistical significance was set at *P* <0.05.

## Results

### Patient demographics and characteristics

Of the 593 patients included in this study, the average age at which the patients underwent TIPS placement was 56 ± 12 years; 272 patients (45.9%) were women. The mean pre-TIPS and post-TIPS MELD scores were 14 ± 7 and 15 ± 7, respectively. Alcoholic liver disease (*n *=* *200; 33.7%) and non-alcoholic fatty-liver disease (*n *=* *165; 27.8%) were the most common etiologies of liver disease among study patients. The two most common indications for TIPS placement were ascites (*n *=* *300; 50.6%) and variceal bleeding (*n *=* *269; 45.4%) (of note, the indications for TIPS placement and the etiologies of liver disease were not mutually exclusive). The mean PSG decreased from 18 ± 6 mmHg pre TIPS to 6 ± 3 mmHg post TIPS placement (*P *<* *0.001; **Table 1**).

**Table 1.** Summary of patient characteristics among 593 patients who underwent TIPS placement

Characteristic	Value
Mean age at time of TIPS placement ± SD, years	56 ± 12
Sex, *n* (%)	
Male	321 (54.1)
Female	272 (45.9)
African American, *n* (%)	40 (6.7)
Presence of hypertension, *n* (%)	208 (35.1)
Pre-TIPS MELD score, mean ± SD (range)	14 ± 7
Post-TIPS MELD score, mean ± SD (range)	15 ± 7
History of encephalopathy, *n* (%)	209 (35.2)
Indication for TIPS placement, *n* (%)^a^	
Ascites	300 (50.6)
Variceal bleeding	269 (45.4)
Hydrothorax	92 (15.5)
Other	44 (7.4)
Emergency TIPS placement, *n* (%)	118 (19.9)
Etiology of liver disease, *n* (%)^a^	
Hepatitis B	14 (2.4)
Hepatitis C	116 (19.6)
Alcoholic liver disease	200 (33.7)
Non-alcoholic steatohepatitis	165 (27.8)
Cryptogenic causes	43 (7.3)
Unknown	84 (14.2)
PSG, mean ± SD (mmHg)	
Pre-TIPS placement	18 ± 6
Post-TIPS placement	6 ± 3
Creatinine level, mean ± SD (mg/dL)	
Pre-TIPS placement	1.26 ± 0.98
Post-TIPS placement	1.11 ± 0.84

TIPS, transjugular intra-hepatic portosystemic shunt; SD, standard deviation; PSG, portosystemic gradient.

a Subgroups are not mutually exclusive.

### Creatinine-level change after TIPS placement

The mean Cr levels at T0, T1, T2, and T3 for all patients were 1.26 ± 0.98, 1.22 ± 1.00, 1.22 ± 0.93, and 1.11 ± 0.84 mg/dL, respectively. The average pre-TIPS Cr level was 0.92 ± 0.26 mg/dL for patients with normal renal function (*n *=* *466; 78.6%) and 2.51 ± 1.49 mg/dL for patients with baseline renal dysfunction (*n *=* *127; 21.4%).

**Figure 1.**
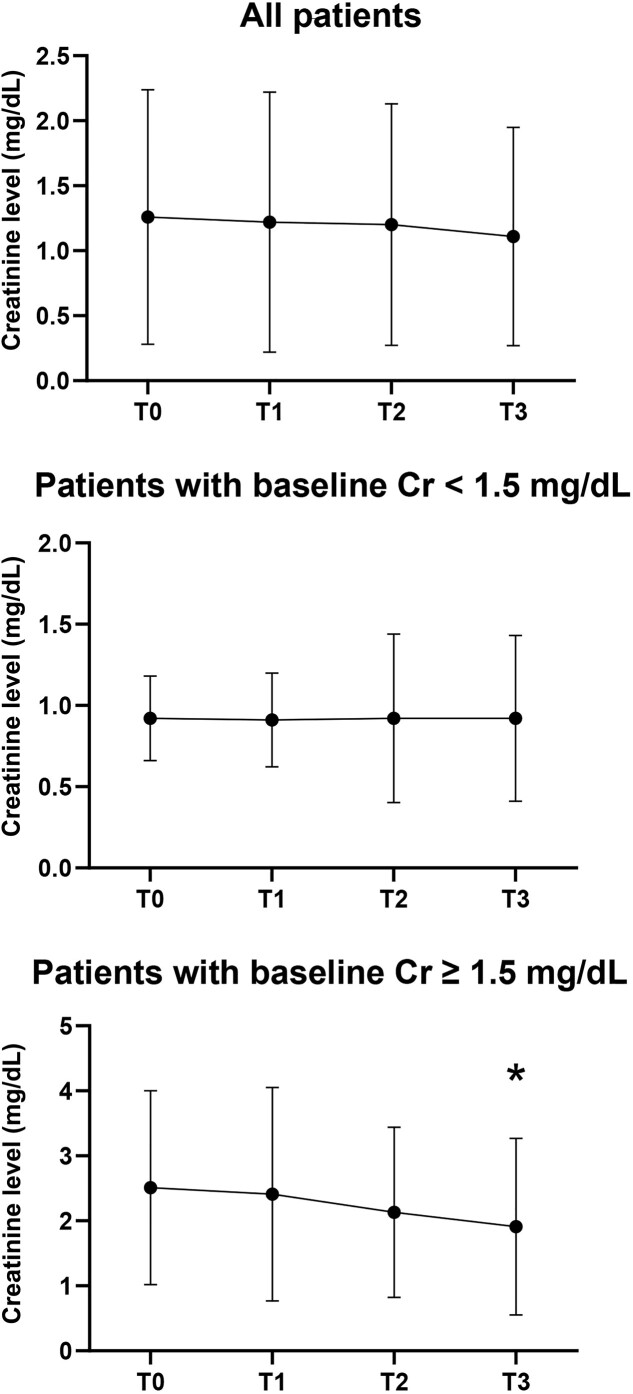
Creatinine (Cr) change from pre-transjugular intra-hepatic portosystemic shunt (TIPS) placement to post-TIPS placement. Averaged Cr levels at T0, T1, T2, and T3 for all patients (top), for patients with normal pre-TIPS Cr levels (middle), and for patients with elevated pre-TIPS Cr levels (bottom) are plotted. Cr levels at T0, T1, T2, and T3 were not significantly different across the entire patient cohort. For patients with normal baseline Cr levels, there was no significant difference between T0 creatinine with T1, T2, or T3 Cr levels. For patients with elevated baseline Cr levels, T3 Cr levels (1.91 ± 1.36 mg/dL) were significantly lower than T0 Cr levels (2.51 ± 1.49 mg/dL; *P *<* *0.001). *Indicates statistical significance.

Overall, the Cr level did not significantly decrease from T0 to T3 (difference, −0.15 mg/dL, −11.9%; *P > *0.05; **Figure 1**). For patients with normal baseline Cr levels, the Cr level did not significantly change from T0 to T3 (difference, <0.01 mg/dL, <0.10% change; **Figure 1**). In contrast, for patients with baseline renal dysfunction, the Cr levels significantly decreased from 2.51 ± 1.49 mg/dL at T0 to 1.91 ± 1.36 mg/dL at T3 (difference, −0.60 mg/dL, −23.9%; *P *<* *0.001; **Figure 1**). There was no significant change from T0 to T1 or from T0 to T2 for patients with normal baseline Cr levels or patients with baseline renal dysfunction.

The presence of hypertension was found to be correlated with a lower degree of Cr-level improvement after TIPS placement (slope = 0.154; *P *=* *0.029; **Table 2**). The etiology of liver disease, history of encephalopathy, indication for TIPS placement, and emergency TIPS placement were not significant predictors of a Cr-level change for the entire patient cohort. A higher pre-TIPS Cr level was significantly correlated with a greater decrease in the Cr level from T0 to T3 (slope = −0.458; *P *<* *0.001; **Table 2**).

**Table 2.** Potential predictors of a creatinine-level decrease after TIPS placement

Predictor	Slope	95% CI	*P*-value
Age (increase of 1 year)	0.003	−0.0004 to 0.009	0.406
Male sex	−0.013	−0.143 to 0.118	0.850
African American	0.221	−0.056 to 0.499	0.119
Hypertension	0.154	−0.560 to 0.292	0.029^*^
History of encephalopathy	−0.052	−0.196 to 0.093	0.480
Etiology of liver disease			
Hepatitis B	−0.156	−0.550 to 0.238	0.439
Hepatitis C	−0.115	−0.293 to 0.063	0.206
Alcoholic liver disease	0.048	−0.110 to 0.205	0.551
Non-alcoholic steatohepatitis	0.015	−0.169 to 0.198	0.876
Indication for TIPS placement			
Ascites	0.024	−0.131 to 0.178	0.764
Variceal bleeding	0.033	−0.134 to 0.201	0.695
Hydrothorax	0.082	−0.096 to 0.260	0.367
Emergency TIPS placement	−0.062	−0.249 to 0.125	0.517
Change in PSG (increase of 1 mmHg)	−0.001	−0.001 to 0.011	0.852
Pre-TIPS creatinine level (increase of 1 mg/dL)	−0.458	−0.523 to 0.392	<0.001^*^

TIPS, transjugular intra-hepatic portosystemic shunt; CI, confidence interval; PSG, portosystemic gradient.

* Indicates statistical significance.

### Mortality risk after TIPS placement

**Figure 2.**
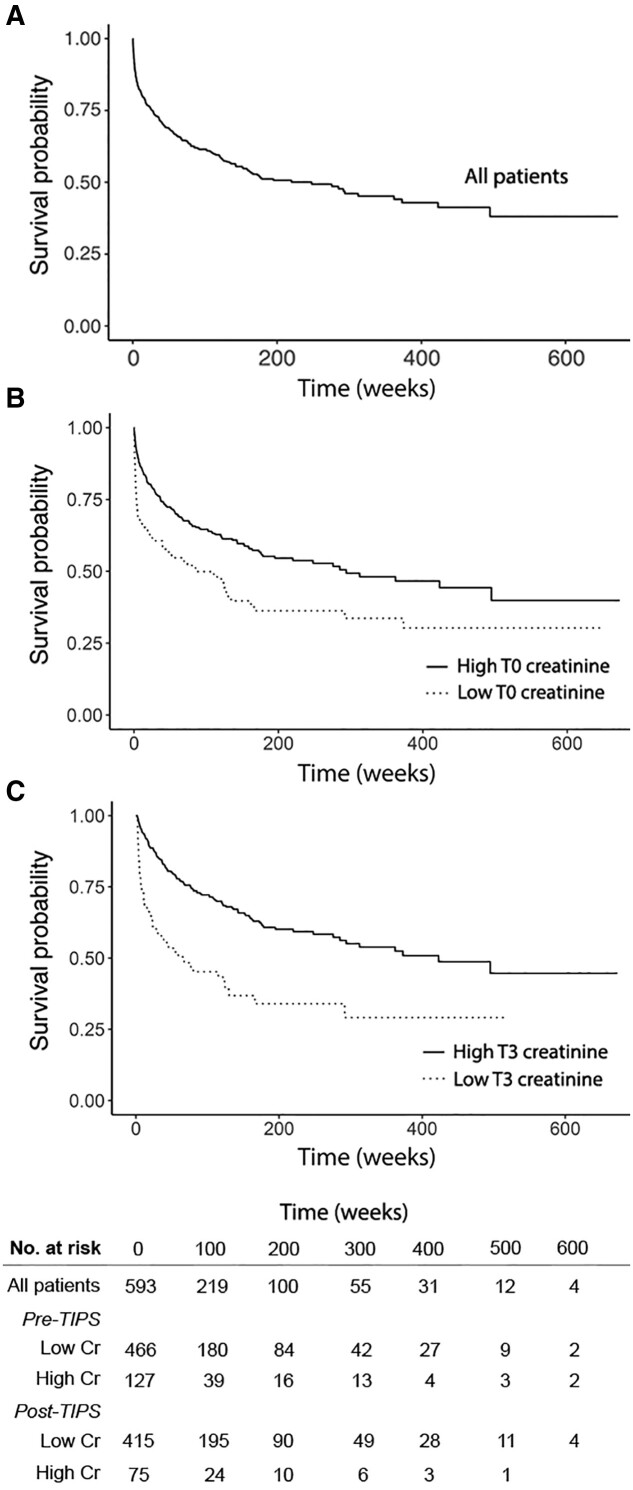
Kaplan–Meier survival plot for all patients (top), for patients subdivided based on creatinine (Cr) levels before transjugular intra-hepatic portosystemic shunt (TIPS) placement (T0) (middle), and for patients subdivided based on Cr levels after TIPS placement (T3) (bottom). Of note, only 490 patients had T3 collected due to either death prior to collection or being lost to follow-up. Survival probability was significantly different between patients with high pre-TIPS Cr levels and those with low pre-TIPS Cr levels (*P *=* *0.0001). Survival probability was also significantly different between patients with high post-TIPS Cr levels and those with low post-TIPS Cr levels (*P *<* *0.0001). The number of patients at risk is indicated in the table at the bottom of the figure.

The 30-day, 90-day, and 1-year mortality rates in our entire patient cohort were 13%, 20%, and 32%. Patients with an elevated T0 Cr level had a significantly higher mortality risk than patients with a normal T0 Cr level (*P *<* *0.001; **Figure 2**). Similarly, patients with higher Cr levels (≥1.5 mg/dL) after TIPS placement (T3) had a significantly higher mortality risk than patients with normal T3 Cr levels (*P *<* *0.001; **Figure 2**).

Variceal bleeding as a TIPS-placement indication (hazard ratio = 1.731; *P *=* *0.036), higher T0 Cr level (hazard ratio = 1.835; *P *=* *0.012), and higher T3 Cr level (hazard ratio = 3.524; *P *<* *0.001) were associated with a higher 1-year mortality risk (**Table 3**). Alcoholic liver disease was significantly associated with a lower 1-year mortality risk (hazard ratio = 0.572; *P *=* *0.039). Of note, the interaction term between the pre-TIPS (T0) Cr level and the post-TIPS (T3) Cr level was significant (*P *=* *0.003), meaning that the effect of the post-TIPS Cr level on mortality risk was dependent on the T0 Cr value and vice versa. There was no significant association between the 1-year mortality risk and age, sex, history of encephalopathy, PSG change, or emergency TIPS placement (*P *>* *0.05 for all).

**Table 3.** Potential predictors of 1-year mortality risk

Predictor	Hazard ratio	95% CI	*P*-value
Age (increase of 5 years)	1.009	0.989–1.030	0.426
Male sex	0.677	0.446–1.027	0.067
African American	0.449	0.142–1.418	0.172
History of encephalopathy	0.976	0.605–1.573	0.919
Hypertension	1.133	0.737–1.741	0.569
Etiology of liver disease			
Hepatitis B	0.392	0.053–2.873	0.357
Hepatitis C	1.047	0.599–1.830	0.871
Alcoholic liver disease	0.572	0.336–0.973	0.039^*^
Non-alcoholic steatohepatitis	0.871	0.497–1.527	0.631
Indication for TIPS placement			
Ascites	1.397	0.870–2.247	0.169
Variceal bleeding	1.731	1.036–2.893	0.036^*^
Hydrothorax	1.629	0.976–2.717	0.062
Emergency TIPS placement	1.022	0.567–1.842	0.943
Change in PSG (increase of 1 mmHg)	1.007	0.966–1.049	0.756
Pre-TIPS (T0) creatinine level (increase of 1 mg/dL)	1.835	1.141–2.951	0.012^*^
Post-TIPS (T3) creatinine level (increase of 1 mg/dL)	3.524	2.306–5.385	<0.001^*^
*Interaction between T0 Cr and T3 Cr levels*	*0.686*	*0.534–0.882*	*0.003*

TIPS, transjugular intra-hepatic portosystemic shunt; CI, confidence interval; PSG, portosystemic gradient.

* Indicates statistical significance.

## Discussion

Many patients with chronic liver disease have concomitant renal dysfunction; however, the exact incidence is unclear and likely to be underestimated [25–27]. Previous studies have demonstrated that renal-function impairment in patients with chronic liver disease is significantly associated with increased rates of morbidity and mortality [25]. The purpose of our study was, therefore, to assess the effect of TIPS placement on renal function and to determine the relationship between the post-TIPS Cr level and the mortality rate. In our large, single-center, retrospective study, we found that TIPS placement significantly improved the renal function in patients with baseline renal dysfunction but did not have a significant impact on patients with normal baseline Cr levels. Furthermore, we found that the post-TIPS Cr level was a strong predictor of 1-year mortality risk and may be an important prognostication factor for post-TIPS survival.

Interestingly, we found that patients with an elevated Cr level at baseline had an overall decrease in the Cr level after TIPS placement, whereas patients with a normal baseline Cr level exhibited almost no change after the procedure. The theory that patients with more severe baseline renal dysfunction may benefit more from TIPS placement has been proposed previously [28]. It has been thought that TIPS placement improves renal function and glomerular filtration by decreasing the release of renin, aldosterone, and noradrenaline [29–33]—an effect that will not have a great impact on cirrhotic patients without baseline renal dysfunction. Furthermore, the presence of hypertension may indicate long-standing renal disease, which can chronically alter renin-angiotensin-system physiology. This may explain why a history of hypertension was inversely correlated with renal improvement and a Cr-level change in our study (**Table 2**). Renal dysfunction itself is currently not an indication for TIPS placement. Our findings, however, suggest that TIPS placement may have a potential role in the preservation of renal function, especially for patients with disease that is nonresponsive to medical management.

The baseline Cr level is a component of the MELD score and its association with mortality risk has been well elucidated. The association between the post-TIPS Cr level and mortality risk, however, has not been previously described. In this study, the T3 Cr level had the strongest effect on post-TIPS survival and patients with a high T3 Cr level (Cr ≥1.5 mg/dL) had a significantly higher risk of mortality than patients with a normal T3 Cr level (<1.5 mg/dL; **Table 3**). While pre-TIPS parameters are commonly investigated for potential predictors of survival, post-TIPS predictors of survival are also important, as these factors can be used to identify high-risk patients who may require closer follow-up or the incorporation of more intensive treatments [34–38]. Our findings suggest that close monitoring of post-TIPS Cr levels is indicated, as these measurements may act as a potential warning to clinicians of worsening disease severity.

TIPS placement has been shown to improve survival in subgroups of cirrhotic patients such as those with acute variceal bleeding or refractory ascites, especially when these patients are treated early in the disease course [39]. In our study, patients with greater baseline renal dysfunction demonstrated a greater decrease in Cr levels after TIPS placement and lower post-TIPS Cr levels were associated with a lower mortality risk. Taken together, these results suggest that TIPS placement may improve survival in patients with more severe renal dysfunction at baseline. This raises the question of whether earlier TIPS placement in cirrhotic patients with signs of worsening renal function can lead to improvement in survival. Cirrhotic patients with a greater degree of renal dysfunction may benefit more from the procedure, but whether there is an upper Cr-level limit beyond which would be related to worse outcomes is unclear. Further investigation is needed to assess the use of increasing pre-TIPS Cr levels as an indication for TIPS placement and the use of post-TIPS Cr levels to identify high-risk patients.

The overall 1-year mortality rate in our study cohort was 27%. Previously reported 1-year mortality rates have varied widely, ranging from 31% to 80% [11, 29, 31]. This wide range of reported mortality rates may be partially explained by studies’ including patients with various levels of disease severity and different institutions using different algorithms for treatment and management. Previous studies have reported that emergency TIPS placement, age, and the presence of ascites before TIPS placement were associated with early TIPS survival [34, 35, 40]. In our multivariable analysis, age, sex, presence of hypertension, history of encephalopathy, change in PSG, and emergency TIPS placement were not significantly associated with post-TIPS mortality. One explanation for this is that we used a multivariable model adjusting for multiple characteristics, whereas some previous studies used unadjusted analysis, which does not take into account possible confounding effects [34, 35]. In addition, population characteristics were heterogeneous across studies, which likely also contributed to the different findings.

TIPS placement is indicated for the management of various conditions, including variceal bleeding, ascites, hepatorenal syndrome, portal hypertensive gastropathy, Budd–Chiari syndrome, hepatic hydrothorax, hepatopulmonary syndrome, and portal-vein thrombosis [41]. Some of the commonly reported indications are associated with a higher post-TIPS mortality rate, include variceal bleeding and portal gastropathy [35–37, 42]. Consistently with previous reports, our analysis demonstrated that variceal bleeding was significantly associated with a greater 1-year mortality risk (hazard ratio of 1.73). Methods to augment the higher risk associated with acute variceal bleeding are being explored, such as early TIPS placement, in which the procedure is performed within 24–72 hours of variceal-bleed presentation [43–46].

This study had several limitations. The first is the retrospective nature of the study. Second, hydration before and after TIPS placement is known to influence post-TIPS Cr levels. Data on the hydration status prior to TIPS placement among our study patients were not available and so this factor may be a confounder in our models. In addition, other factors such as renal-function drugs and the frequency of paracentesis performed for ascites were unavailable and were not included for analysis. Finally, because of incomplete follow-up, TIPS-associated complications such as hepatic encephalopathy were unable to be assessed.

In summary, cirrhotic patients with baseline renal dysfunction demonstrated a significant improvement in renal function after TIPS placement, whereas patients with normal renal function exhibited no overall change. Post-TIPS Cr levels had the strongest effect on 1-year mortality risk, suggesting that the monitoring of post-TIPS Cr levels may be helpful in identifying high-risk patients who may require closer follow-up or the incorporation of more intense treatments.

## Authors’ contributions

M.L., W.W., B.K.E., B.S., and B.K. conceived and designed the project. M.L., A.L.L., B.Q.T., and B.K.E. collected the data. M.L., A.L.L., B.Q.T., and B.K. analysed and interpreted the data. M.L., A.L.L., W.W., B.S., and B.K. drafted the manuscript. All authors read and approved the final manuscript.

## Funding

None.
